# Les tumeurs malignes primitives de l’intestin grêle: aspects cliniques et thérapeutiques de 27 patients

**Published:** 2011-03-03

**Authors:** Halima Abahssain, Maha Mokrim, Issam Lalya, Hind M’rabti, Hassan Errihani

**Affiliations:** 1 Service d’oncologie médicale, Institut national d’oncologie, Rabat, Maroc; 2 Service de radiothérapie, Institut national d’oncologie, Rabat, Maroc

**Keywords:** Intestin grêle, tumeur maligne, diagnostic, chirurgie, chimiothérapie, survie, Maroc

## Abstract

Les tumeurs malignes de l’intestin grêle (TMPIG) sont des tumeurs rares. Elles représentent 1 à 5% de toutes les tumeurs du tube digestif. Elles sont caractérisées par une hétérogénéité anatomopathologique et une symptomatologie pauvre et non spécifique entrainant ainsi un retard diagnostic, des difficultés Thérapeutiques et donc un mauvais pronostic. Nous rapportant les caractéristiques épidémiologiques, diagnostiques et thérapeutiques ainsi que la survie des patients atteints des TMIG au sein de l’institut national d’oncologie de Rabat. Il s’agit d’une analyse rétrospective des dossiers cliniques des 27 patients ayant le diagnostic de tumeurs malignes de l’intestin grêle admis dans notre institut entre 1998 et 2002. L’âge médian était de 46 ans (15-70 ans). Le délai médian de diagnostic était de 6 mois (0-96 mois). La douleur abdominale était le symptôme le plus fréquent (77.8%). L’étude anatomopathologique a montré 63% de lymphome non Hodgkinien, 14.8% d’adénocarcinome, 7.4% de tumeur stromale, 7.4% de carcinome neuroendocrine et 7.4% de sarcome intestinal. Vingt patients (76. 9%) ont eu une résection chirurgicale et 14 patients (53. 8%) ont eu une chimiothérapie en fonction du stade de la maladie et du type histologique. Après un recul médian de 11.6 mois, la survie globale après 12 mois était de 44.4% et la médiane de survie était de 11.6 mois. Les tumeurs malignes de l’intestin grêle sont rares. Leur diagnostic est tardif limitant ainsi la prise en charge thérapeutique curative. Les cliniciens doivent être avertis des symptômes gastro-intestinaux non spécifiques.

## Introduction

Les tumeurs malignes primitives de l’intestin grêle (TMPIG) sont des tumeurs rares. Elles représentent 1 à 5% de toutes les tumeurs du tube digestif bien que l’intestin grêle représente 75 % de la longueur totale et plus de 90% de la surface muqueuse du tractus digestif [1-3].

Selon une revue de plus de 11 000 tumeurs primaires malignes gastro-intestinales publiées par Martin et all, seulement 2.4%, 10.8%, 16.4% et 70.3% des tumeurs malignes primitives sont respectivement d’origine grêlique, œsophagienne, gastrique, colique et rectale [4].

La multiplicité des types histologiques associée à la rareté des de ces tumeurs et l’absence d’essais randomisés prospectives élucidant les meilleures options diagnostiques et thérapeutiques rendent difficile d’établir des statistiques valables.

L’intestin grêle est considéré comme une zone cliniquement silencieuse et il en résulte un retard diagnostic et donc un traitement non optimal et un pronostic sévère. Nous rapportant les caractéristiques épidémiologiques, diagnostiques et thérapeutiques ainsi que la survie des patients atteints des tumeurs de l’intestin grêle au sein de l’institut national d’oncologie de Rabat au Maroc.

## Méthodes

Il s’agit d’une analyse rétrospective des dossiers cliniques de 27 patients ayant le diagnostic de TMPIG admis dans notre institut entre 1998 et 2002. Le comité scientifique de l’institut national d’oncologie a approuvé l’analyse rétrospective des dossiers des patients inclus dans cette étude. Tous les patients ayant une confirmation histologique d’une tumeur maligne du duodénum jusqu’à l’iléon terminal ont été inclus dans l’étude.

Le diagnostic de TMPIG a été posé sur une étude anatomopathologique soit de la pièce de résection chirurgicale intestinale ou sur une biopsie tumorale guidée par le scanner ou par l’échographie. Les bilans diagnostiques ont été demandés en fonction de la localisation de la tumeur et de sa nature histopathologique. Le traitement de chaque patient a été décidé en réunion de concertation pluridisciplinaire en fonction du type histologique de la tumeur, de sa localisation, de son extension à distance et du tableau clinique initial. Les patients ont reçu soit une chirurgie ou une chimiothérapie ou les deux armes thérapeutiques. La chirurgie a consisté à des résections segmentaire en fonction du siège et de l’étendu de la tumeur.

Les protocoles de chimiothérapie utilisés étaient soit le protocole FUFOL (Acide folique à 20 mg/m2/j, puis 5Fluo-uracile à 425 mg/m2/j en bolus IV de J1 à J5 tous les 28 jours), le protocole AF ( doxorubicine,5Fluorouracile), le protocole COP( cyclophosphamide à 750 mg/m^2^, vincristine à 1,4mg/ m^2^ sans dépasser la dose totale de 2 mg par cure, prednisone à 40mg/m^2^ pendant 5 jours), le protocole CHOP(cyclophosphamide à 750 mg/m^2^, doxorubicine à 50mg/m^2^, vincristine à 1,4mg/ m^2^ sans dépasser la dose totale de 2 mg par cure, prednisone à 40mg/m^2^ pendant 5 jours), ou le cyclophosphamide seule en continue à la dose de 25mg/m^2^ per os).

**Suivi **

Les patients étaient suivis jusqu’au mois de janvier 2010. Ceux qui n’ont pas étaient revus à la dernière consultation étaient contactés par téléphone.

**Analyse statistique**

L’analyse statistique a été réalisée en utilisant le programme statistique pour les sciences sociales (SPSS) version13. Les statistiques descriptives ont été utilisées pour étudier les caractéristiques des patients. La survie globale a été calculée par la méthode de Kaplan-Meier.

**Consentement et approbation éthique **

Chaque patient a été traité par l’équipe médicale de l’institut national d’oncologie de Rabat après avoir obtenu un consentement verbal et validation de la décision thérapeutique par la réunion de concertation pluridisciplinaire. Notre étude a été approuvée par le comité étique de l’institut national d’oncologie à Rabat.

## Résultats

**Figure 1:**
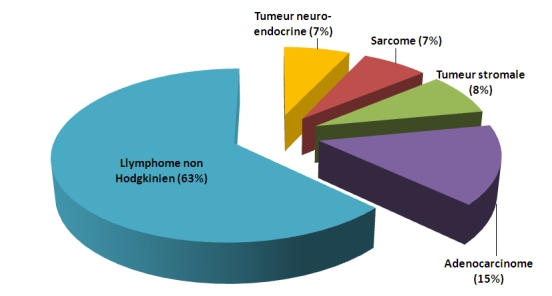
Répartition des tumeurs malignes primitives de l’intestin grêle en fonction du type histologique chez un groupe de 27 patients atteints des tumeurs de l’intestin grêle à l’institut national d’oncologie de Rabat au Maroc entre 1998 et 2002

Parmi les 27 patients colligé au sein de l’institut, 19 étaient des hommes (70.4%) et 8 étaient des femmes (29.6%). L’âge médian était de 46 ans (15 - 70 ans). Le délai médian entre le début de la symptomatologie et le diagnostic était de 6 mois (0 - 96 mois). La douleur abdominale était présente chez 77.8% (21 patients), une masse abdominale dans 37% (10 patients), et l’occlusion intestinale inaugurale dans 37% (10 patients). L’endoscopie digestive a été réalisée dans 24% des cas (6 patients), l’opacification digestive dans 24% (6 patients) et la tomodensitométrie abdominale dans 60%. L’étude anatomopathologique a montré que 63% de ces tumeurs intestinales étaient un lymphome non Hodgkinien (LNH) (17 patients), un adénocarcinome (ADK) dans 14.8% des cas (4 patients), une tumeur stromale dans 7.4% des cas (2 patients), une tumeur carcinoïde dans 7.4% des cas (2 patients) et un sarcome intestinal dans 7.4% des cas (2 patients) (Figure 1).

**Figure 2:**
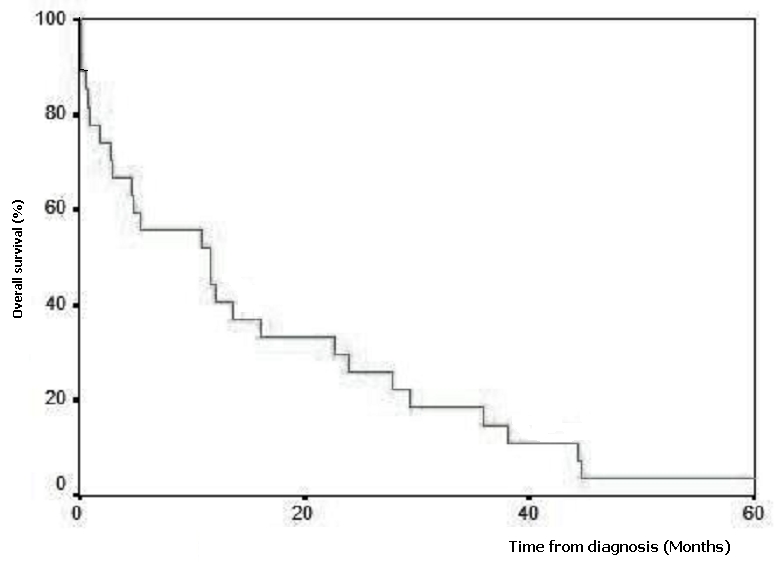
Courbe de survie d’un groupe de 27 patients atteints des tumeurs de l’intestin grêle à l’institut national d’oncologie de Rabat au Maroc entre 1998 et 2002

Le suivi médian était de 11.6 mois. La survie globale après 12 mois était de 44.4% et la médiane de survie était de 11.6 mois (Figure 2).

Sur les 17 patients ayant un LNH grêlique, 11 patients (68,8%) ont eu une résection chirurgicale dont 8 en urgence après une occlusion intestinale aigüe. Onze patients avec un LNH ont reçu une chimiothérapie dont 6 ont reçu un protocole CHOP, quatre un protocole COP et un patient du cyclophosphamide per os. La survie à un an des patients avec un LNH grêlique est de 29.4%.

Pour les quatre patients avec un ADK grêlique, trois d’entre eux ont eu une résection chirurgicale dont un en urgence suite à une occlusion intestinale aigüe. Deux patients avaient une maladie localisée et ont reçu une chimiothérapie adjuvante type FUFOL mayo clinique. Les deux autres patients avec un ADK métastatique n’ont pas reçu de chimiothérapie à cause d’un index de Karnofsky bas.

Les 2 patients avec une tumeur stromale ont eu une résection chirurgicale seule. Les 2 patients avec une tumeur neuroendocrine ont eu une résection chirurgicale dont un en état d’urgence suite à une occlusion intestinale aigüe. L’autre patient a reçu 6 cures de chimiothérapie à base de 5fluoro-uracile et la doxorubicine. Les deux patients avec un sarcome intestinal ont eu une résection chirurgicale seule et ont décédé à 23 et 45 mois respectivement.

## Discussion

Les tumeurs malignes de l’intestin grêle sont des tumeurs rares [4-9]. Leur carcinogénèse n’est pas bien claire. Néanmoins, leur incidence est particulièrement faible en raison de la régénération rapide de la muqueuse de l’intestin grêle, la faible densité bactérienne qui produits des métabolites carcinogènes, la rapidité du transit qui réduit le temps de contact de certains agents carcinogènes et le taux élevé de l’hydroxylase benzopyren qui neutralise l’effet des carcinogènes ce qui diminue les probabilités de genèse d’un cancer. En outre l’importance du tissu lymphoïde au niveau de l’intestin grêle ainsi que la forte concentration des IgA au niveau iléale protège l’intestin grêle contre les virus et empêche la croissance tumorale [10-12].

Les tumeurs de l’intestin grêle sont caractérisées par une symptomatologie clinique non spécifique à cause de sa grande distensibilité et de son contenu liquidien, ce qui rend leur diagnostic difficile et la maladie est souvent découvert à un stade avancé [3]. Plusieurs auteurs ont rapporté que les douleurs abdominales étaient parmi les symptômes les plus retrouvés dans cette localisation tumorale.

Dans notre série les douleurs abdominales étaient le symptôme révélateur le plus fréquent [5-7,13,14]. Catena et al. ont rapporté les aspects cliniques des tumeurs grêliques et ils ont montré qu’elles peuvent souvent se manifester par des urgences abdominales telles que l’occlusion intestinale aigüe [15]. Dans notre série 37% des patients ont été diagnostiqués suite à une urgence abdominale.

Le taux des tumeurs malignes de l’intestin grêle est de 64% approximativement dont 40% sont des adénocarcinomes [14]. Selon une série de Hatzaras et al [5], les tumeurs carcinoïdes du grêle sont les tumeurs les plus fréquentes suivies par les ADK, cependant dans notre série les LNH grêliques sont les tumeurs malignes les plus fréquentes suivies par les adénocarcinomes. La résection chirurgicale des ADK grêliques localisés restent la seule option pour la guérison [9,16]. Elle peut également être indiquée au stade de la maladie localement avancée pour pallier aux symptômes ou lors des urgences abdominales [16]. Plusieurs auteurs ont rapporté le bénéfice d’une chimiothérapie dans les tumeurs malignes de l’intestin grêle, cependant le protocole de chimiothérapie optimale ainsi que la vraie définition de ce bénéfice doit être élucidé [17-19].

Actuellement l’association de 5-fluorouracile à un sel de platine semble être la combinaison la plus efficace en situation adjuvante et palliative en dépit de l’absence d’études prospectives randomisées [20].

Dans notre série seulement 4 patients avaient un ADK grêlique dont deux étaient localisés et ont reçu une chimiothérapie adjuvante. Les deux autres patients avec une maladie métastatique n’ont pas reçu de chimiothérapie à cause de l’altération de l’état général.

Beaucoup de controverse persiste concernant le rôle du traitement chirurgicale dans le traitement des LNH grêlique localisés (stade I et II) [21]. En effet la plupart des informations disponibles sur l’efficacité de la chirurgie dans les LNH de l’intestin grêle au stade localisé sont basées sur des études restreintes rétrospectives [22,23]. La question à laquelle devraient répondre des études rigoureuses est la nécessité ou pas d’une chimiothérapie post opératoire après une résection complète d’un LNH de l’intestin grêle localisé [24]. Le traitement des patients avec un LNH grêlique avancé (stade III et IV) est actuellement calqué sur celui des LNH ganglionnaire, est basé sur un ensemble d’options thérapeutiques (chimiothérapie, thérapie ciblées, immunothérapie, greffe de cellules souches hématopoïétiques)[25].

Dans notre série plus de 68% de patients avec un LNH grêlique ont eu une résection chirurgicale soit dans le cadre d’une urgence abdominale chirurgicale ou comme un geste curatif. La chimiothérapie reçue par ces patients était calquée sur celle des LNH ganglionnaires et les protocoles variaient en fonction de la disponibilité des produits de la chimiothérapie dans l’institut hospitalière.

Le pronostic des tumeurs malignes de l’intestin grêle est très sévère. Il est lié à leurs symptomatologie non spécifique, au retard diagnostic du à des difficultés diagnostic, à la présence d’une extension locorégionale et à distance lors du diagnostic et à la présence de plus de 70% de localisation péritonéale ou à distance au moment de la chirurgie [26,27].

Dans la série de Howe et al la médiane de survie des patients avec une tumeur duodénale, jéjunale et iléale était respectivement de 16.9, 28 et 31 mois [19]. Dans notre série la médiane de survie de nos patients était de 11.6 mois.

Selon l’étude de Han SL et al, le siège tumoral initial, le stade tumoral et le type histologique n’influence pas la survie [28]. Ito et al ont rapporté que le taux de survie à 5 ans des patients avec un T1/T2 et T3/T4 est de 82% et 58% respectivement (p<0.05) [29]. Cependant Bakaeen et al ont trouvé que le stade T ne peut pas prédire la survie des patients atteintes de tumeur malignes du grêle [18].

Notre étude est rétrospective, elle comporte plusieurs limites qui peuvent biaiser l’interprétation des résultats. Les 2 limitations majeures sont d’une part, l’absence dans certains dossiers exploités des données sur la stadification de la maladie et d’autre part, la non disponibilité de certains produits de chimiothérapie ou de thérapie ciblée dans notre institut au moment de la prise en charge des patients de cette étude.

## Conclusion 

Les TMPIG sont des tumeurs rares. Le diagnostic est difficile à cause de la symptomatologie non spécifique. Le pronostic reste sévère vue la découverte de la maladie à un stade avancé. La prise en charge de ces tumeurs malignes dépend du type histologique et reste un défit pour l’équipe soignante. Des essais randomisés prospectifs devraient apporter des réponses sur la prise en charge diagnostique, thérapeutique et par conséquent améliorer le pronostic de nos patients.

## Remerciements

Les auteurs remercient l’équipe du service d’épidémiologie et des archives de l’institut national d’oncologie de Rabat de nous avoir fourni les données nécessaires pour la réalisation de ce travail.

## Conflits d’intérêt

Les auteurs déclarent l’absence de conflit d’intérêt.

## Contribution des auteurs

HA a conçu l’étude, exploité les données, effectué l’analyse statistique et rédigé le manuscrit. MM et LI ont participé à la saisie et à l’exploitation des données. HM et HE ont révisé le manuscrit. Tous les auteurs ont lu et approuvé la version finale du manuscrit.
